# Season, Sex, Age, and Education as Modifiers of the Effects of Outdoor Air Pollution on Daily Mortality in Shanghai, China: The Public Health and Air Pollution in Asia (PAPA) Study

**DOI:** 10.1289/ehp.10851

**Published:** 2008-07-09

**Authors:** Haidong Kan, Stephanie J. London, Guohai Chen, Yunhui Zhang, Guixiang Song, Naiqing Zhao, Lili Jiang, Bingheng Chen

**Affiliations:** 1 Department of Environmental Health, School of Public Health, Fudan University, Shanghai, China; 2 Epidemiology Branch, National Institute of Environmental Health Sciences, National Institutes of Health, U.S. Department of Health and Human Services, Research Triangle Park, North Carolina, USA; 3 Shanghai Environmental Monitoring Center, Shanghai, China; 4 Shanghai Municipal Center of Disease Control and Prevention, Shanghai, China; 5 Department of Health Statistics, School of Public Health, Fudan University, Shanghai, China

**Keywords:** air pollution, modifiers, mortality, time-series studies

## Abstract

**Background:**

Various factors can modify the health effects of outdoor air pollution. Prior findings about modifiers are inconsistent, and most of these studies were conducted in developed countries.

**Objectives:**

We conducted a time-series analysis to examine the modifying effect of season, sex, age, and education on the association between outdoor air pollutants [particulate matter < 10 μm in aerodynamic diameter (PM_10_), sulfur dioxide, nitrogen dioxide, and ozone] and daily mortality in Shanghai, China, using 4 years of daily data (2001–2004).

**Methods:**

Using a natural spline model to analyze the data, we examined effects of air pollution for the warm season (April–September) and cool season (October–March) separately. For total mortality, we examined the association stratified by sex and age. Stratified analysis by educational attainment was conducted for total, cardiovascular, and respiratory mortality.

**Results:**

Outdoor air pollution was associated with mortality from all causes and from cardiorespiratory diseases in Shanghai. An increase of 10 μg/m^3^ in a 2-day average concentration of PM_10_, SO_2_, NO_2_, and O_3_ corresponds to increases in all-cause mortality of 0.25% [95% confidence interval (CI), 0.14–0.37), 0.95% (95% CI, 0.62–1.28), 0.97% (95% CI, 0.66–1.27), and 0.31% (95% CI, 0.04–0.58), respectively. The effects of air pollutants were more evident in the cool season than in the warm season, and females and the elderly were more vulnerable to outdoor air pollution. Effects of air pollution were generally greater in residents with low educational attainment (illiterate or primary school) compared with those with high educational attainment (middle school or above).

**Conclusions:**

Season, sex, age, and education may modify the health effects of outdoor air pollution in Shanghai. These findings provide new information about the effects of modifiers on the relationship between daily mortality and air pollution in developing countries and may have implications for local environmental and social policies.

Epidemiologic studies have reported associations of outdoor air pollution with daily mortality and morbidity from cardiorespiratory diseases (Goldberg et al. 2003). Multicity analyses conducted in the United States, Canada, and Europe provide further evidence supporting coherence and plausibility of the associations (Burnett et al. 2000; Dominici et al. 2006; Katsouyanni et al. 1997, 2001; Samet et al. 2000a). Recently, interest has been focused on the possible modifying effect of season (Peng et al. 2005; Touloumi et al. 2006; Zeka et al. 2006), preexisting health status (Bateson and Schwartz 2004; Goldberg et al. 2001; Katsouyanni et al. 2001), and population demographic characteristics such as sex and age (Atkinson et al. 2001; Bateson and Schwartz 2004; Cakmak et al. 2006; Katsouyanni et al. 2001) on the relation between air pollution and daily mortality. It is also hypothesized that the effects of air pollution exposure on health are greater in people with lower socioeconomic status (SES) (O’Neill et al. 2003). However, prior findings about the modifying effect of SES remain inconsistent: some studies found evidence of modification (Finkelstein et al. 2003; Jerrett et al. 2004; Krewski et al. 2005; Zeka et al. 2006), but others did not (Bateson and Schwartz 2004; Cakmak et al. 2006; Samet et al. 2000b; Zanobetti and Schwartz 2000). Moreover, most of these studies were conducted in developed countries, and only a small number of studies have been conducted in Asia (Health Effects Institute 2004). The need remains for studies of cities in developing countries, where characteristics of outdoor air pollution (e.g., air pollution level and mixture, transport of pollutants), meteorological conditions, and sociodemographic patterns may differ from those in North America and Europe.

Better knowledge of these modifying factors will help in public policy making, risk assessment, and standard setting, especially in cities of developing countries with fewer existing studies. In the present study, we conducted a time-series analysis to examine the modifying effect of season, sex, age, and education on the association between outdoor air pollutants [particulate matter < 10 μm in diameter (PM_10_), sulfur dioxide, nitrogen dioxide, and ozone] and daily mortality in Shanghai, China. This study is a part of the joint Public Health and Air Pollution in Asia (PAPA) program supported by the Health Effects Institute (HEI).

## Materials and Methods

### Data

Shanghai, the most populous city in China, comprises urban/suburban districts and counties, with a total area of 6,341 km^2^ and had a population of 13.1 million by the end of 2004. Our study area was limited to the traditional nine urban districts of Shanghai (289 km^2^). The target population includes all permanent residents living in the area—around 6.3 million in 2004. In the target population, the male/female ratio was 100.9%, and the elderly (> 65 years of age) accounted for 11.9% of the total population.

Daily nonaccidental mortality data from 1 January 2001 to 31 December 2004 were collected from the database of the Shanghai Municipal Center of Disease Control and Prevention (SMCDCP). Death certificates are completed either by community doctors for deaths at home or by hospital doctors for deaths in hospitals. The information on the certificates is then sent to the SMCDCP through their internal computer network. In Shanghai, all deaths must be reported to appropriate authorities before cremation. The database for 2001 and 2002–2004 was coded according to the *International Classification of Diseases*, *Revision 9* [ICD-9; World Health Organization (WHO) 1978] and *Revision 10* (ICD-10; WHO 1993), respectively. The mortality data were classified into deaths due to all nonaccidental causes (ICD-9 codes < 800; ICD-10 codes A00–R99), cardiovascular diseases (ICD-9 codes 390–459; ICD-10 codes I00–I99), and respiratory diseases (ICD-9 codes 460–519; ICD-10 codes J00–J98). The data were also classified by sex and age (0–4, 5–44, 45–64, and ≥ 65 years) for all-cause deaths. Educational attainment has often been used as a surrogate indicator of SES in time-series studies (Cakmak et al. 2006; Jerrett et al. 2004; Zanobetti and Schwartz 2000; Zeka et al. 2006). We therefore classified all-cause, cardiovascular, and respiratory deaths by educational attainment (low, illiterate or primary school; high, middle school or above).

Daily air pollution data, including PM_10_, SO_2_, NO_2_, and O_3_, were retrieved from the database of the Shanghai Environmental Monitoring Center, the government agency in charge of collection of air pollution data in Shanghai. The daily concentrations for each pollutant were averaged from the available monitoring results of six fixed-site stations in the nine urban districts and covered by China National Quality Control. These stations are mandated to be located away from major roads, industrial sources, buildings, or residential sources of emissions from the burning of coal, waste, or oil; thus, our monitoring results reflect the background urban air pollution level in Shanghai rather than local sources such as traffic or industrial combustion.

We abstracted the daily 24-hr mean concentrations for PM_10_, SO_2_, and NO_2_, and maximal 8-hr mean concentrations for O_3_. The maximal 8-hr mean was used because the WHO (2000) recommended that the 8-hr mean reflects the most health-relevant exposure to O_3._ For the calculation of both 24-hr mean concentrations of PM_10_, SO_2_, and NO_2_, as well as maximal 8-hr mean O_3_ concentrations, at least 75% of the 1-hr values must have been available on that particular day.

To allow adjustment for the effect of weather conditions on mortality, we obtained daily mean temperature and humidity data from the Shanghai Meteorological Bureau database. The weather data were measured at a single fixed-site station in the Xuhui District of Shanghai.

All of the mortality, weather, and air pollution data were validated by an independent auditing team assigned by the HEI. The team checked a sample of the original death certificates and monitoring records and validated the generation process of mortality, weather, and air pollution data used for the time-series analysis.

### Statistical methods

Our statistical analysis followed the Common Protocol of the PAPA program. We used a generalized linear model (GLM) with natural splines (ns) to analyze the data. First, we built the basic models for various mortality outcomes excluding the air pollution variables. We incorporated the ns functions of time and weather conditions, which can accommodate nonlinear and non-monotonic relationships of mortality with time and weather variables, offering a flexible modeling tool (Hastie and Tibshirani 1990). We used the partial autocorrelation function (PACF) to guide the selection of degrees of freedom (df) for time trend (Katsouyanni et al. 2001; Touloumi et al. 2004, 2006). Specifically, we used 4–6 df per year for time trend. When the absolute magnitude of the PACF plot was < 0.1 for the first two lag days, the basic model was regarded as adequate; if this criterion was not met, autoregression terms for lag up to 7 days were introduced to improve the model. In this way, 4, 4, and 5 df per year for time trend, as well as 3, 2, and 4 lag-day autoregression terms, were used in our basic models for total, cardiovascular, and respiratory mortality, respectively. In addition, we used 3 df (whole period of study) for temperature and humidity because this has been shown to control well for their effects on mortality (Dominici et al. 2006; Samet et al. 2000a). Day of the week was included as a dummy variable in the basic models. We examined residuals of the basic models to determine whether there were discernable patterns and autocorrelation by means of residual plots and PACF plots. After we established the basic models, we introduced the pollutant variables and analyzed their effects on mortality outcomes.

Briefly, we fit the following log-linear GLM to obtain the estimated pollution log-relative rate β in Shanghai:


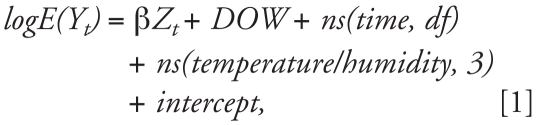


where *E(Y_t_)* represents the expected number of deaths at day *t*; β represents the log-relative rate of mortality associated with a unit increase of air pollutants; *Z_t_* indicates the pollutant concentrations at day *t; DOW* is dummy variable for day of the week; *ns(time,df)* is the ns function of calendar time; and *ns(temperature/humidity, 3)* is the ns function for temperature and humidity with 3 df. Current-day temperature and humidity (lag 0) and 2-day moving average of air pollutant concentrations (lag 01) were used in our analyses.

We assessed both total nonaccidental and cause-specific mortality. We were able to stratify by sex and age only for total mortality. We analyzed effects of air pollution separately for the warm season (April–September) and the cool season (October–March) as well as for the entire year (Peng et al. 2005; Touloumi et al. 2006). The basic models of seasonal analyses were different from those of whole-period analyses, using various dfs for time trend. Analyses by educational attainment were conducted for total, cardiovascular, and respiratory mortality. We tested the statistical significance of differences between effect estimates of the strata of a potential effect modifier (e.g., the difference between females and males) by calculating the 95% confidence interval (CI) as


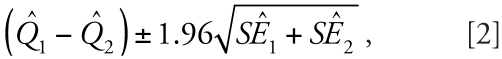


where Q̂_1_ and Q̂_2_ are the estimates for the two categories, and *S*Ê_1_ and *S*Ê_2_ are their respective SEs (Zeka et al. 2006). Regardless of significance, we considered modification of effect by a factor of ≥ 2 to be important and worthy of attention (Zeka et al. 2006).

As a sensitivity analysis, we also examined the impact of model specifications such as lag structure and df selection on the effects of air pollutants (Welty and Zeger 2005). We did not find substantial differences using alternative specifications.

All analyses were conducted in R, version 2.5.1, using the mgcv package (R Development Core Team 2007). The results are presented as the percent change in daily mortality per 10-μg/m^3^ increase of air pollutants.

## Results

### Data description

From 2001 to 2004 (1,461 days), a total of 173,911 deaths (82,597 females and 91,314 males) were registered in the study population. The percentages of total deaths by age group were 0.3% for 0–4 years, 3.2% for 5–44 years, 13.0% for 45–64 years, and 83.5% for ≥ 65 years. On average, there were approximately 119 nonaccidental deaths per day, including 44 from cardiovascular diseases and 14 from respiratory diseases (Table 1). Cardiorespiratory disease accounted for 49.1% of total nonaccidental deaths.

During our study period, the mean daily average concentrations of PM_10_, SO_2_, NO_2_, and O_3_ were 102.0, 44.7, 66.6, and 63.4 μg/m^3^, respectively. There were two missing value days for O_3_ and none for the other three pollutants. The mean daily average temperature and humidity were 17.7°C and 72.9%, respectively, reflecting the subtropical climate in Shanghai.

Generally, PM_10_, SO_2_, and NO_2_ were relatively highly correlated with each other (Pearson correlation coefficients ranged from 0.64 to 0.73). PM_10_/SO_2_/NO_2_ concentrations were negatively correlated with temperature and humidity. Maximal 8-hr mean O_3_ was weakly correlated with PM_10_, SO_2_, and NO_2_ (Pearson correlation coefficients ranged from 0.01 to 0.19) and moderately correlated with temperature level (Pearson correlation coefficient, 0.48).

### Effects by season

In the whole-period analyses, outdoor air pollution was associated with mortality from all causes and from cardiopulmonary diseases in Shanghai (Table 2). An increase of 10 μg/m^3^ of 2-day average concentrations of PM_10_, SO_2_, NO_2_, and O_3_ corresponds to 0.25% (95% CI, 0.14–0.37), 0.95% (95% CI, 0.62–1.28), 0.97% (95% CI, 0.66–1.27), and 0.31% (95% CI, 0.04–0.58) increase of all-cause mortality, respectively.

There were more deaths, higher concentrations of pollutants (except for O_3_, which had higher concentrations in the warm season), and drier weather conditions in the cool season than in the warm season (Table 1).

The effect estimates of PM_10_ on total mortality were similar in both seasons. Effect estimates were approximately 2–3 times higher for SO_2_ and NO_2_ in the cool season compared with the warm season. The effect estimate of O_3_ was significant in both cool and warm seasons, and the magnitude of the O_3_-associated increase in total mortality was approximately 5-fold higher in the cool season than in the warm season. Between-season differences in total mortality were significant for NO_2_ and O_3_ but not for PM_10_ or SO_2_ (Table 2).

For cardiovascular mortality, the effect estimate of PM_10_ was similar in both seasons. For SO_2_, NO_2_, and O_3_, the effect estimate in the cool season were approximately 3–4 times higher than in the warm season. Between-season differences in cardiovascular mortality were insignificant for all four pollutants.

For the smaller category of respiratory mortality, the effect estimates of PM_10_, SO_2_, and NO_2_ were significant only in the cool season, and their between-season differences were significant. The effect effect estimate of O_3_ on respiratory mortality was insignificant in either season.

### Effects by sex and age

The percent increase associated with higher concentration levels of air pollutants varied by sex or age group (Table 3). The effect estimates of PM_10_ and O_3_ among females were approximately twice those among males, although their between-sex differences were insignificant. The effect estimates of SO_2_ and NO_2_ on total mortality in females were slightly higher than in males.

The number of deaths for residents under 5 years of age was very low and therefore was excluded from our analysis. We did not observe significant effects of air pollution in residents 5–44 years of age or 45–64 years of age. Among those ≥ 65 years of age, the effect estimates of all four pollutants were significant, and approximately 2–5 times higher than among people 5–44 years of age or 45–64 years of age, although the between-age differences among all three groups were insignificant.

### Effects by education

Generally, residents with low educational attainment (illiterate or primary school) had a higher number of deaths from air pollution–related effects than those with high educational attainment (middle school or above) (Table 4).

For total mortality, the effect estimates of PM_10_, SO_2_, and NO_2_ were significant in both education groups. The effect estimates of these three pollutants were 1–2 times larger among the low-education group compared with the high-education group, although the educational differences were significant only for NO_2_ for total mortality. The effect estimate of O_3_ of total mortality were similar and insignificant in both groups.

For cardiovascular mortality, the effect estimates of PM_10_ and NO_2_ were significant or marginally significant in both education groups; the effect estimate of SO_2_ was significant only in the low-education group; no significant effect of O_3_ was seen in either group. The effect estimates of all four pollutants were 1–2 times larger among the low-education group compared with the high-education group. The educational differences in cardiovascular mortality were not significant for any pollutants.

For respiratory mortality, the effect estimates of PM_10_, SO_2_, and NO_2_ were significant only among those with low education, whereas the effect estimate of O_3_ on respiratory mortality was not significant in either group. The effect estimates of PM_10_, SO_2_, and NO_2_ were several times larger among the low-education group compared with the high-education group. The educational differences in respiratory mortality were not significant for any pollutants.

## Discussion

Although the associations between outdoor air pollution and daily mortality have been well established in developed countries, the question of the potential modifiers remains inconclusive. As the U.S. National Research Council (1998) pointed out, it is important to understand the characteristics of individuals who are at increased risk of adverse events due to outdoor air pollution. Our results suggest that season and individual sociodemographic factors (e.g., sex, age, SES) may modify the health effects of air pollution in Shanghai. Specifically, the association between air pollution and daily mortality was generally more evident for the cool season than the warm season; females and the elderly (≥65 years of age) appeared to be more vulnerable to air pollution than males and younger people; and disadvantaged SES may intensify the adverse health effects of outdoor air pollution.

Our finding of a stronger association between air pollution and daily mortality in the cool season is consistent with several prior studies in Hong Kong (Wong et al. 1999, 2001) and Athens, Greece (Touloumi et al. 1996), but in contrast with others reporting greater effects in the warm season (Anderson et al. 1996; Bell et al. 2005; Nawrot et al. 2007). In Shanghai, the concentrations of PM_10_, SO_2_, and NO_2_ were higher and more variable in the cool season than in the warm season (Table 1). Because these three pollutants were highly correlated, greater effects observed during the cool season may also be due to other pollutants that were also at higher levels during that season. In contrast, the O_3_ level was higher in the warm season than in the cool season, and our exposure–response relationship also revealed a flatter slope at higher concentrations of O_3_ for both sexes (data not shown). At higher concentrations, the risks of death could be reduced because vulnerable subjects may have died before the concentration reached the maximum level (Wong et al. 2001).

Exposure patterns may contribute to our season-specific observation. During the warm season, Shanghai residents tend to use air conditioning more frequently because of the relatively higher temperature and humidity, thus reducing their exposure. For example, in a survey of 1,106 families in Shanghai, 32.7% of the families never turn on air conditioners in the winter compared with 3.7% in the summer (Long et al. 2007). Heavy rain in the warm season may reduce time outdoors, thus reducing personal exposure. In contrast, the cool season in Shanghai is drier and less variable, so people are more likely to go outdoors and open the windows. Nevertheless, the fact that a consistently significant health effect of air pollution was observed only in the cool season in two subtropical Asian cities [Shanghai (present study) and Hong Kong (Wong et al. 1999, 2001)] suggests that the interaction of air pollution exposure and season may vary by location.

Unlike the gaseous pollutants, the constituents of the complex mix of PM_10_ may vary by season. Therefore, another potential explanation for the seasonal difference in the effects of PM_10_ is that the most toxic particles may have a cool-season maximum in Shanghai.

We found a greater effect of ambient air pollution on total mortality in females than in males. Results of prior studies on sex-specific acute effects of outdoor air pollution were discordant. For example, Ito and Thurston (1996) found the highest risk of mortality related with air pollution exposure among black women. Hong et al. (2002) found that elderly women were most susceptible to the adverse effects of PM_10_ on the risk of acute mortality from stroke. However, Cakmak et al. (2006) found that sex did not modify the hospitalization risk of cardiac diseases due to air pollution exposure.

The reasons for our sex-specific observations are unclear and deserve further investigation. In Shanghai, females have a much lower smoking rate than males (0.6% in females vs. 50.6% in males) (Xu 2005). One study suggested that effects of air pollution may be stronger in nonsmokers than in smokers (Künzli et al. 2005). Oxidative and inflammatory effects of smoking may dominate to such an extent that the additional exposure to air pollutants may not further enhance effects along the same pathways in males. In addition, females have slightly greater airway reactivity than males, as well as smaller airways (Yunginger et al. 1992); therefore, dose–response relations might be detected more easily in females than in males. Deposition of particles in the lung varies by sex, with greater lung deposition fractions of 1-μM particles in all regions for females (Kim and Hu 1998; Kohlhaufl et al. 1999). Sunyer et al. (2000) suggested that differing particulate deposition patterns between females and males may partly explain the difference between the sexes. Moreover, compared with males, females in Shanghai had a lower education level (73.9% in females vs. 41.0% in males); thus, lower SES might contribute to the observed larger effects of air pollution in females.

As in a few other studies (Gouveia and Fletcher 2000; Katsouyanni et al. 2001), we found the elderly were most vulnerable to the effects of air pollution. Low numbers of deaths in the 0- to 4-year age group limited our power to detect the effects of air pollution on mortality, even if they exist. Two groups, the elderly and the very young, are presumed to be at greater risk for air pollution–related effects (Gouveia and Fletcher 2000; Schwartz 2004). For the elderly, preexisting respiratory or cardiovascular conditions are more prevalent than in younger age groups; thus, there is some overlap between potentially susceptible groups of older adults and people with heart or lung diseases.

It has long been known that SES can affect health indicators such as mortality (Mackenbach et al. 1997). Recently, studies have started to examine the role of SES in the vulnerability of subpopulations to outdoor air pollution, especially for particles and O_3_, although the results remain inconsistent (O’Neill et al. 2003). For example, Zeka et al. (2006) found that individual-level education was inversely related to the risk of mortality associated with PM _10 ._Another cohort study with small-area measures of SES in Hamilton, Ontario, Canada, found important modification of the particle effects by social class (Finkelstein et al. 2003; Jerrett et al. 2004). In contrast, Gouveia and Fletcher (2000) observed a larger effect of air pollution in areas of higher SES level; Bateson and Schwartz (2004) found no indication that susceptibility to air pollution varied by group-level SES measures. In the present study, using individual-level education as a measure of SES, we found that residents with low educational attainment were more sensitive to air pollution exposure than those with high educational attainment. Our results provide the first evidence in Mainland China that lower SES may compose a risk factor for air pollution–related health effects.

SES factors such as educational attainment may modify the health effects of outdoor air pollution in several pathways. People with lower SES may be more sensitive to air pollution–related health hazards because they have a higher prevalence of preexisting diseases that confer a greater risk of dying associated with air pollution exposure, and they may also receive inferior medical treatment for preexisting diseases. Disadvantaged living conditions may contribute to the modification effect; people with lower SES may have more limited access to fish, fresh fruits, and vegetables, resulting in reduced intake of antioxidant polyunsaturated fatty acids and vitamins that may protect against adverse consequences of particle exposure (Romieu et al. 2005). Additionally, exposure patterns may contribute to effect modification by SES. Persons with lower SES are less likely to have air conditioning (Long et al. 2007) and more likely to live near busy roadways and have coexposures due to either poor housing or occupation. For example, disadvantaged groups have been found to be more highly exposed to some air pollutants (Sexton et al. 1993). Scandinavian studies have shown differential personal exposures to particles and other pollutants by education and occupation (Rotko et al. 2000, 2001), and a study in the U.S. Great Lakes region indicates differences in exposure to gaseous pollutants by occupation and education, minority status, and income (Pellizzari et al. 1999). Finally, as Jerrett et al. (2004) pointed out, persons with lower education are less mobile and experience less exposure measurement error, thereby reducing bias toward the null.

The limitations of our analysis should be noted. As in other studies in this field, we used available outdoor monitoring data to represent the population exposure to air pollutants. Our assessment of weather conditions was derived entirely from one monitoring station. Measurement error may have substantial implications for interpreting epidemiologic studies on air pollution, particularly for the time-series design (Zeger et al. 2000). It is possible that this type of error may introduce bias to the results of our analysis; however, because of lack of available information on personal exposure to air pollutants, we could not quantify such a bias. Compared with other studies in Europe and North America, the data we collected were limited in being only one city, in sample size, and in duration. In addition, high correlation between particulate matter and gaseous pollutants in Shanghai limited our ability to separate the independent effect for each pollutant.

In summary, in this time-series analysis, we found that outdoor air pollution was associated with mortality from all causes and from cardiopulmonary diseases in Shanghai during 2001–2004. Furthermore, our results suggest that season and sociodemographic factors (e.g., sex, age, SES) may modify the acute health effects of air pollution. These findings provide new information about the effects of modifiers on the relationship between daily mortality and air pollution in developing countries and may have implications for local environmental and social policies.

## Figures and Tables

**Table 1:** Daily deaths, air pollutant concentrations, and weather conditions (mean ± SE) in Shanghai, China, 2001–2004.

	Warm season (*n* = 729)	Cool season (*n* = 732)	Entire period (*n* = 1,461)
No. of daily deaths			
Total (nonaccident)	106.1 ± 0.5	132.0 ± 0.8	119.0 ± 0.6
Cardiovascular	37.9 ± 0.3	50.5 ± 0.4	44.2 ± 0.3
Respiratory	11.4 ± 0.1	17.2 ± 0.3	14.3 ± 0.2
Air pollutant concentration (μg/m^3^)^a^			
PM_10_	87.4 ± 1.8	116.7 ± 2.8	102.0 ± 1.7
SO_2_	39.4 ± 0.7	50.1 ± 1.0	44.7 ± 0.6
NO_2_	57.3 ± 0.7	76.0 ± 1.0	66.6 ± 0.7
O_3_	78.4 ± 1.5	48.3 ± 0.9	63.3 ± 1.0
Meteorological measures			
Temperature (°C)	24.3 ± 0.2	11.2 ± 0.3	17.7 ± 0.2
Humidity (%)	75.1 ± 0.4	70.6 ± 0.5	72.9 ± 0.3

a Twenty-four-hour average for PM_10_, SO_2_, and NO_2_; 8-hr (1000–1800 hours) average for O_3_.

**Table 2:** Percent increase [mean (95% CI)] of mortality outcomes of Shanghai residents associated with 10-μg/m^3^ increase in air pollutant concentrations by season, 2001–2004.^a^

Mortality	Pollutant	Warm season	Cool season	Entire period
Total	PM_10_	0.21 (0.09 to 0.33)	0.26 (0.22 to 0.30)	0.25 (0.14 to 0.37)
	SO_2_	0.57 (−0.03 to 1.18)	1.10 (0.66 to 1.53)	0.95 (0.62 to 1.28)
	NO_2_	0.46 (−0.07 to 0.98)	1.24 (0.84 to 1.64)*	0.97 (0.66 to 1.27)
	O_3_	0.22 (0.03 to 0.41)	1.19 (0.56 to 1.83)*	0.31 (0.04 to 0.58)
Cardiovascular	PM_10_	0.22 (−0.14 to 0.58)	0.25 (0.05 to 0.45)	0.27 (0.10 to 0.44)
	SO_2_	0.31 (−0.65 to 1.29)	1.02 (0.40 to 1.65)	0.91 (0.42 to 1.41)
	NO_2_	0.30 (−0.54 to 1.14)	1.26 (0.68 to 1.84)	1.01 (0.55 to 1.47)
	O_3_	0.32 (−0.05 to 0.69)	1.42 (0.51 to 2.33)	0.38 (−0.03 to 0.80)
Respiratory	PM_10_	−0.28 (−0.93 to 0.38)	0.58 (0.25 to 0.92)*	0.27 (−0.01 to 0.56)
	SO_2_	−1.13 (−2.86 to 0.62)	2.47 (1.41 to 3.54)*	1.37 (0.51 to 2.23)
	NO_2_	−1.37 (−2.86 to 0.15)	2.66 (1.67 to 3.65)*	1.22 (0.42 to 2.01)
	O_3_	0.12 (−0.72 to 0.98)	0.94 (−0.60 to 2.50)	0.29 (−0.44 to 1.03)

a We used current day temperature and humidity (lag 0) and 2-day moving average of air pollutant concentrations (lag 01), and applied 3 df to temperature and humidity.

* Significantly different from the warm season (p < 0.05).

**Table 3:** Percent increase [mean (95% CI)] in total mortality of Shanghai residents associated with a 10-μg/m^3^ increase in air pollutant concentrations by sex and age.^a^

		Pollutant			
	Mean daily deaths (*n*)	PM_10_	SO_2_	NO_2_	O_3_
Sex					
Female	56.5	0.33 (0.18 to 0.48)	1.06 (0.62 to 1.51)	1.10 (0.69 to 1.51)	0.40 (0.03 to 0.76)
Male	62.5	0.17 (0.03 to 0.32)	0.85 (0.43 to 1.28)	0.88 (0.49 to 1.28)	0.19 (−0.16 to 0.55)
Age (years)					
5–44	3.7	0.04 (−0.52 to 0.59)	1.21 (−0.47 to 2.91)	0.52 (−1.01 to 2.08)	−0.08 (−1.38 to 1.25)
45–64	15.5	0.17 (−0.11 to 0.45)	0.22 (−0.60 to 1.04)	0.64 (−0.11 to 1.40)	0.47 (−0.19 to 1.12)
≥65	99.6	0.26 (0.15 to 0.38)	1.01 (0.65 to 1.36)	1.01 (0.69 to 1.34)	0.32 (0.03 to 0.61)

a We used current day temperature and humidity (lag 0) and 2-day moving average of air pollutant concentrations (lag 01), and applied 3 df to temperature and humidity.

**Table 4:** Percent increase in number of deaths due to total, cardiovascular, and respiratory causes associated with a 10-μg/m^3^ increase in air pollutants by educational attainment.^a^

			Pollutant			
Mortality	Educational attainment	Mean daily deaths (*n*)	PM_10_	SO_2_	NO_2_	O_3_
Total	Low	67.3	0.33 (0.19 to 0.47)	1.19 (0.77 to 1.61)	1.27* (0.89 to 1.66)	0.26 (−0.09 to 0.60)
	High	42.1	0.18 (0.01 to 0.36)	0.66 (0.16 to 1.17)	0.62 (0.15 to 1.09)	0.30 (−0.11 to 0.71)
Cardiovascular	Low	27.8	0.30 (0.10 to 0.51)	1.08 (0.47 to 1.69)	1.15 (0.58 to 1.72)	0.39 (−0.13 to 0.90)
	High	16.4	0.23 (−0.03 to 0.50)	0.57 (−0.20 to 1.35)	0.73 (0.01 to 1.45)	0.26 (−0.38 to 0.91)
Respiratory	Low	8.9	0.36 (0.00 to 0.72)	1.54 (0.43 to 2.66)	1.59 (0.57 to 2.62)	0.20 (−0.74 to 1.16)
	High	5.4	0.02 (−0.43 to 0.47)	0.73 (−0.61 to 2.09)	0.34 (−0.89 to 1.60)	0.27 (−0.86 to 1.41)

a We used current day temperature and humidity (lag 0) and 2-day moving average of air pollutants concentrations (lag 01) and we applied 3 df to temperature and humidity.

* Significantly different from high educational attainment (p < 0.05).
